# Improvement of appointment compliance in an underserved lupus clinic

**DOI:** 10.1186/s12913-018-3429-7

**Published:** 2018-08-06

**Authors:** Anand Kumthekar, Beverly Johnson

**Affiliations:** 1 Division of Rheumatology, Montefiore Medical Center/ Albert Einstein College of Medicine, 1776 Eastchester Road, Fl. 2, Suite 260, Bronx, NY 10461 USA; 2 Division of Rheumatology, Jacobi Medical Center/Albert Einstein College of Medicine, 1400 Pelham Parkway, 3N21, Bronx, NY 10461 USA

**Keywords:** Clinic show rate, Systemic lupus erythematosus (SLE), Telephone reminder, Underserved clinic

## Abstract

**Background:**

To identify major obstacles to appointment compliance and quantify a measurable effect of a simple phone call intervention on the clinic show rate.

**Methods:**

We retrospectively looked at the show rates from November 1st, 2013 to June 30th, 2014 at our Lupus clinic, which is located in Bronx, NY. The scheduled patient chart was crosschecked if the patient made it to the appointment by verifying the provider note. A patient survey was implemented over a period of 8 weeks from July 1st, 2014 to August 12th, 2014. A reminder phone call intervention 2–3 days prior to the visit was planned. The intervention was implemented from September 1st, 2014 to April 30th, 2015. Data was analyzed after the end of the intervention period.

**Results:**

In the pre-intervention period, our clinic show-rate was 207/352 (58.8%) The pilot survey had a total of 43 responses. The most common reason for a missed appointment was ‘forgot about the appointment’ (45.5%). Reminder phone calls were the preferred intervention (76.74%), which patients’ thought might help to keep scheduled appointments. In the intervention period, 283 of the scheduled 378 appointments were completed (74.8) in the lupus clinic. The difference in the show rate before and after the intervention by Pearson’s Chi-squared test with Yates continuity correction was statistically significant with a *p*-value of 0.0062.

**Conclusion:**

A simple telephone call reminder significantly improves clinic show rates in an underserved Lupus clinic, which can help improve health parameters in the Lupus population.

## Background

Appointment compliance is a nationwide problem with a higher prevalence in underserved communities. For physicians’ no-shows result in lost time, decreased efficiency, and a higher use of resources [1]. For patients, it results in dissatisfaction and reduced quality of care [2]. No-shows cause scheduling and operational difficulties for clinics and can also hamper the patient-provider relationship [3]. The financial losses due to patient no-shows are substantial and prevalent throughout the country [4, 5]. An improved show rate increases the proportion of used appointments and creates an overall decrease in the number of scheduled appointments, which is better for the clinic flow, and continuity of care [6].

Lupus patients are particularly susceptible to bad outcomes if they are lost to follow-up given the disease complexity. There is high morbidity and mortality due to complications such as lupus nephritis, neuropsychiatric lupus and pericardial and pleural effusions. Non-compliance is an important factor in morbidity among lupus patients and missing an appointment for a lupus patient can make the difference in terms of keeping the patient out of the hospital [7].

## Methods

**Fig. 1:**
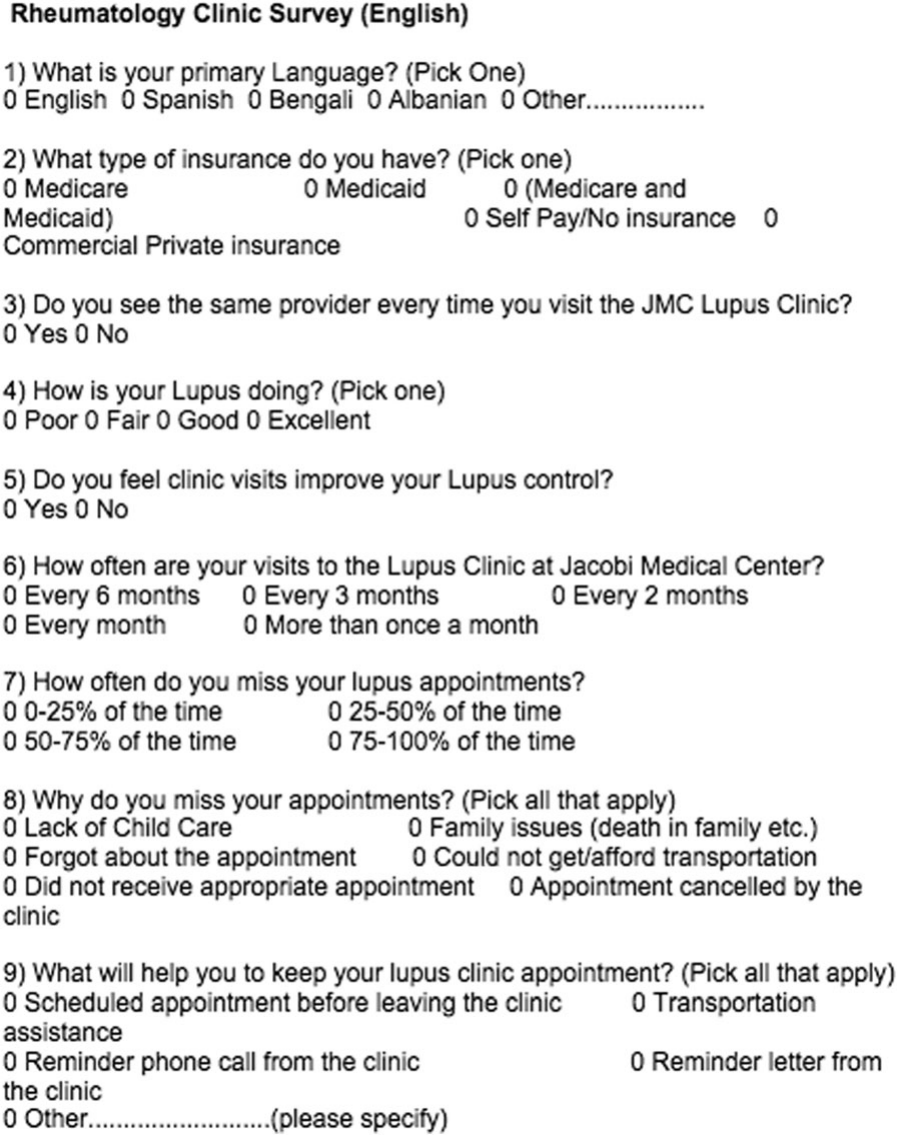
Rheumatology Clinic Survey (English)

We conducted a historical assessment of the total appointments provided and completed at our lupus clinic located at a tertiary care center in Bronx, NY serving a ‘low income’ population with primarily Medicare, Medicaid or no insurance. The pre-intervention phase was defined from November 1st, 2013 to June 30th, 2014. Appointments were confirmed using Sorian scheduling software, and patients were confirmed to have attended their appointment by verifying the provider’s visit note. A pilot questionnaire was designed to understand the barriers to patients attending their lupus clinic appointments and was made available in English and Spanish (Fig. 1). The survey was provided to patients in the waiting room before their appointment. The survey was implemented over a six-week period from July 1st, 2014 to August 12th, 2014 and was collected on the same day. An intervention in the form of a reminder phone call two to three days before the scheduled appointment was implemented in the intervention phase from September 1st, 2014 to April 30th, 2015. A patient clinical associate (PCA) was assigned to make the reminder phone calls. The reminder phone call was made to the best number listed by the patient; either cell phone or land line. A minimum of one attempt from the clinic staff was required. A voicemail was left if there was no answer. The primary objective of the study was to analyze the effect of reminder phone calls on the lupus clinic show rate. Data analysis was done at the end of the study period using Pearson Chi-Square test. Informed consent was not obtained from the participants as no protected health information (PHI) was collected and the study was a practice improvement project. The Institutional Review Board (IRB) at Albert Einstein College of Medicine granted reviewed the project and an IRB exemption was granted per local IRB guidelines.

Inclusion criteria for the pre-intervention and post-intervention analysis were patients who were expected to attend the scheduled lupus clinic appointment. For the survey, we included adult patients over the age of 18 years who attended their lupus clinic appointment between July 1st, 2014 and August 12th, 2014 who agreed to fill the survey in the waiting area before the clinic visit. Patients under the age of 18 years and who were not able or willing to fill out the survey were excluded.

## Results

**Fig. 2:**
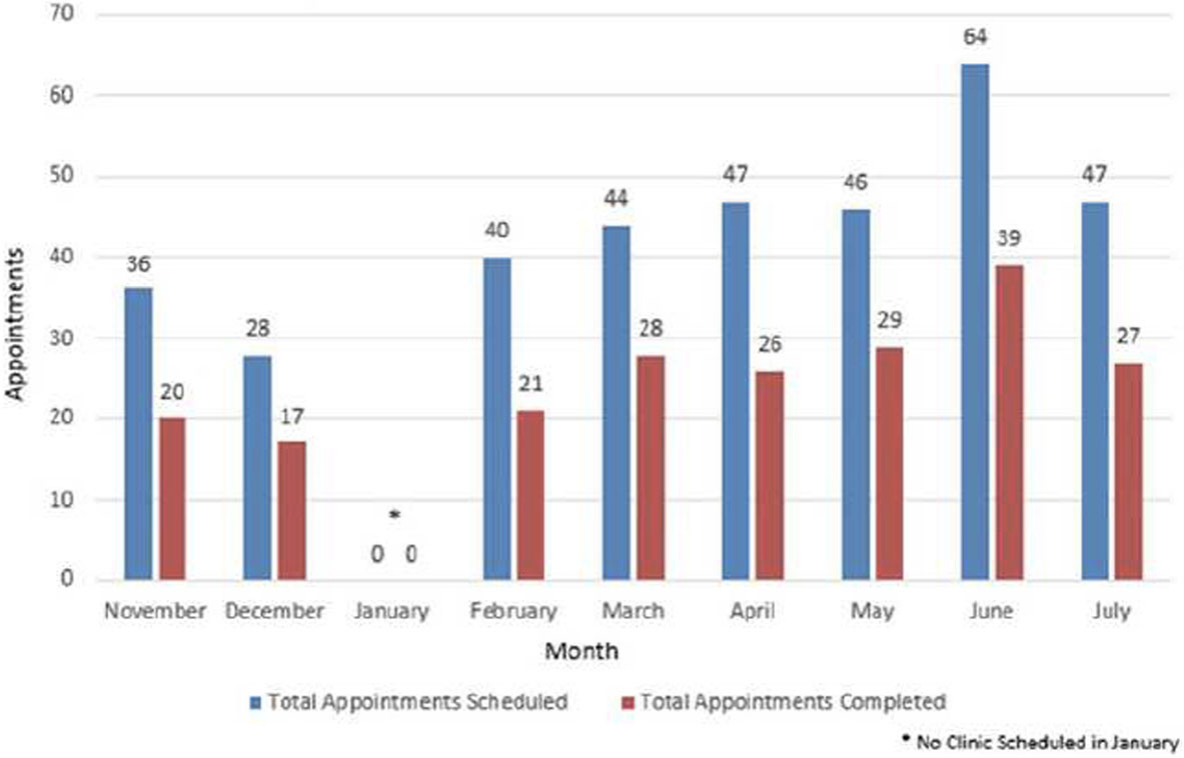
Appointment Compliance (Pre-Intervention phase)

The majority of the patients seen in our clinic either have Medicare, Medicaid or are self-pay. In the pre-intervention period, 352 lupus clinic appointments were scheduled and 207 visits were completed (58.8%) (Fig. 2). The pilot survey had a total of 43 responses. Public insurance was the most common type of insurance 76.7 and 18.6% had no insurance coverage and opted for self-pay. Around 84.7% respondents mentioned they missed under a quarter of their appointments and 13.9% said they missed up to half of their appointments. Reminder phone calls were the preferred intervention (76.7%), which patients’ thought might help to keep scheduled appointments. The most common reason for missed appointment was ‘forgot about the appointment’ (45.5%). This was followed by ‘family issues’ (18.6%), ‘lack of child care’ (14%) or ‘did not receive appropriate appointment’ (14%). Some patients were not able to attend due to lack of transportation related issues (2.3%) and 2 (4.6%) patients reported their last clinic appointment were cancelled by our clinic. Our clinic provides single ride metro card (New York City public transport fare card) for patients who come to the appointment if required. Eligible Medicaid patients had the option of using access a ride (New York City transportation program for people with disabilities who are unable to use public bus or subway service) to come to their appointments.

**Fig. 3:**
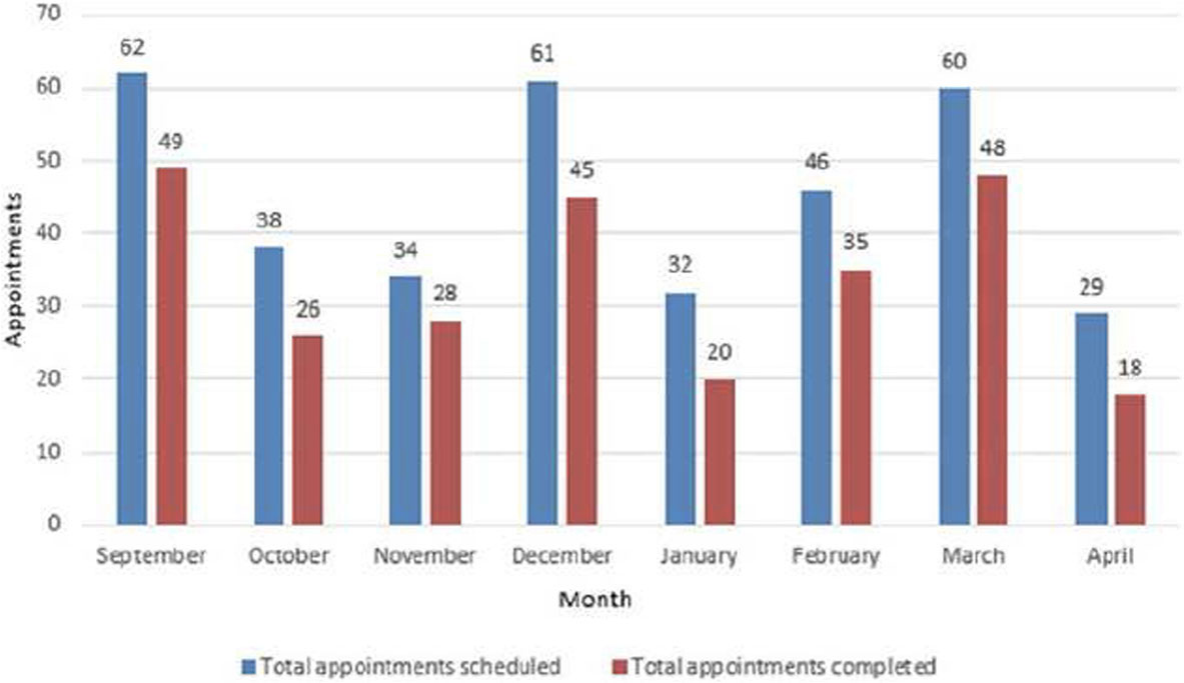
Appointment Compliance (Post-Intervention phase)

In the intervention period, 378 lupus clinic appointments were scheduled and 283 visits were completed (Fig. 3). The difference in the show rate before and after the intervention (58.8% vs. 74.8%) was analyzed using Pearson’s Chi-squared test with Yates’ continuity correction and the *p*-value was 0.0062, which was statistically significant.

## Discussion

In order to improve quality of care for lupus patients in an underserved community in a city hospital, this study attempted to determine barriers to patients attending clinic visits and to design an intervention that could be feasibly implemented. Our study shows that a simple phone call reminder can improve clinic show rates in a lupus clinic located in an underserved community. An improved show rate increases the proportion of used appointments and consequently has an overall decrease in the number of scheduled appointments. This is better for the clinic flow and continuity of care [6].

No notification of the appointment has been widely reported as an important reason for non-compliance across the literature [8]. Different types of phone calls have been tried in other studies ranging from automated phones to the clinic staff calling to text messaging. In an academic outpatient practice, no show rates were significantly lower in patients getting staff or automated phone reminder [9]. A meta-analysis of 23 randomized trials involving adult patients over a 25-year period demonstrated that telephone prompts were consistently useful in reducing broken appointments [10].

Another study showed that telephone reminders are a very cost effective measure in increasing attendance rates in a hospital-based clinic, independent of confounding factors like gender, mode of payment and whether the recipient was the patient, family member or voice mail system [11]. Also, a clinic staff member making reminder phone call adds more importance to the appointment as compared to an automated appointment reminder [12]. A large study demonstrated that apart from telephone calls, a reminder using the messaging service also improves show rates [13]. Our study supports that a reminder phone call from the clinic staff can help with appointment attendance.

It is important to note that there are also other issues that can impact a patient not coming for appointments beyond a reminder phone call. A variety of factors including the perception of their illness, demographic characteristics and socio-economic factors have been associated with visit compliance [8, 14]. The ability to keep a clinic appointment may be enhanced by the types of incentives offered to patients. Incentives like transportation assistance and monetary assistance have been shown to improve compliance [11, 15, 16].

A strength of our study is that it quantified a measurable effect of a simple intervention of a phone call on the show rate in an underserved, low-income community. Some of the limitations of our study include a small sample size and the different times of the year for the pre-intervention and the intervention phase along with the unpaired sample that is studied.

Our study highlights that a small intervention can have significant impact on clinic show rates even in an underserved community with limited resources and other reasons for low show rates. Lupus patients have been shown to have higher morbidity with non-compliance [7] and our hope is that this data can be used by similar clinics to maximize the quality of care for low-income lupus patients who have been shown to be at the highest risk of complications [17].

## Conclusion

Our study suggests that not being able to remember an appointment was a common barrier for missed clinic appointments. A simple telephone call reminder significantly improves clinic show rates in an underserved Lupus clinic, which can help improve health parameters in the Lupus population.

## Abbreviations

IRB: Institutional Review Board
PCA: Patient clinical associate
SLE: Systemic Lupus Erythematosus
